# Lack of BRAF mutations in hyalinizing trabecular neoplasm

**DOI:** 10.1186/1742-6413-3-17

**Published:** 2006-07-25

**Authors:** Zubair W Baloch, Kanchan Puttaswamy, Marcia Brose, Virginia A LiVolsi

**Affiliations:** 1 Departments of Pathology and Laboratory Medicine, 3400 Spruce Street, University of Pennsylvania Medical Center, Philadelphia, PA, 19104, USA; 2 Department of Hematology & Oncology, 3400 Spruce Street, University of Pennsylvania Medical Center, Philadelphia, PA, 19104, USA; 3 Department of Otorhinolaryngology, 3400 Spruce Street, University of Pennsylvania Medical Center, Philadelphia, PA, 19104, USA

## Abstract

The hyalinizing trabecular neoplasm (HTN) of the thyroid is an unusual and controversial lesion. Some consider it a peculiar type of papillary thyroid carcinoma (PTC) because of its nuclear features and presence of psammoma bodies. Others consider it an adenoma. Molecular studies have found RET/PTC translocations in some examples, supporting HTN as a PTC; however mutations in BRAF (another marker for PTC) have not been found.

We report two cases of classic HTN and a case of trabecular PTC and show BRAF mutations in the latter and not in HTN. Trabecular growth pattern is insufficient for a diagnosis of HTN and lesions with such a pattern and nuclear features of PTC are cancers. Morphologically classic HTN are not associated with metastatic potential and should be considered adenomas.

## Background

The hyalinizing trabecular neoplasm (HTN) is a follicular derived lesion that has a distinctive histology. It was first described by Carney and colleagues in 1987 as an unusual tumor of thyroid with benign clinical behavior.[1] Microscopically, these tumors are encapsulated (usually thin capsule) and grow in nests that are surrounded by dense hyaline stroma.[1,2] The histology is reminiscent to that seen in paragangliomas; however, the tumor is derived from the follicular epithelium.[3] The nuclear features of the tumor cells are similar to those seen in papillary thyroid carcinoma (PTC) i.e. elongated nuclei with chromatin clearing, intranuclear grooves and inclusions.[1,4-7] Psammoma bodies have also been reported in HTN.[1] Since its description some experts believe that HTN is an encapsulated variant of PTC based upon its nuclear cytology, immunoprofile and RET-oncogene rearrangements while other believe that this is not a distinct entity because similar growth pattern can be encountered in other primary and secondary tumors of the thyroid. [7-14]

By immunohistochemistry, the cells of hyalinizing trabecular neoplasm stain positive for thyroglobulin, cytokeratin-19 and negative for calcitonin, HBME-1 and galectin-3 although the presence of other neuroendocrine markers has been described[10,15]

RET oncogene encodes the tyrosine kinase (TK) membrane receptor for glial cell line-derived neuron-trophic factors. In PTC chromosomal translocations at 10q11.2 lead to the fusion of the RET TK domain to heterologous genes (RET/PTC oncogenes) and causing activation of its signaling and transforming properties. RET/PTC1 and RET/PTC3 are the most common translocations seen in cases of PTC. RET/PTC translocations have been described in some cases of HTN[11]

The somatic mutations of BRAF gene leading to the substitution of a valine for a glutamic acid (V600E) have been found in 36–69% of PTC and are independent of RET/PTC translocations and RAS gene mutations. To date BRAF mutations have not been detected in HTN. [16-20]

In the present study we report pathologic features and BRAF mutation analysis in 2 cases of HTN and 1 case of trabecular variant of PTC.

## Material and methods

Archival paraffin-embedded tumor samples were retrieved from the pathology files at the University of Pennsylvania Medical Center (2 cases of HTN) and personal consultation files of one of the authors (VAL, 1 case). In all cases hematoxylin and eosin stained slides were available; in one case of HTN fine-needle aspiration slides were also available for review.

### DNA isolation and sequencing

From paraffin slides, five 10 micron thick sections were macro dissected for DNA extraction using Qiagen DNA Mini Kit protocol (Hilden, Germany). The BRAF exon 15 was amplified from genomic DNA obtained from the paraffin samples by polymerase chain reaction (PCR) with the following primers: BRAFex15Forward **5'TCATAATGCTTGCTCTGATAGGA3' **and BRAFex15Reverse **5'GGCCAAAAATTTAATCAGTGGA3'**.

PCR was performed using the following amplification conditions: initial denaturation at 95°C for 15 min followed by 35 cycles of the following steps: denaturation at 95°C for 30 s, annealing at 56.4°C for 30 s, and elongation at 72°C for 30 s. After the last cycle, a final extension at 70°C for 10 minutes was performed. The amplified products were electrophoresed on a 1.2% gel at 110 V for 1.5 hours and the BRAF bands (~220 bp) were cut using sterile blade and purified using Qiagen Gel Extraction Kit (Hilden, Germany). The samples were analyzed on an ABI PRISM 3730 Sequence Analyzer (Applied Biosystems, Foster City, CA, USA). BRAF V600E mutations were confirmed by reamplification by polymerase chain reaction and by sequencing from both 5' and 3' ends. Pooled genomic DNA (showing wild type BRAF sequence) obtained from blood of normal individuals was used as control.

## Results

### Pathologic findings

All three patients were females and the average age was 50 yrs. The tumor size in HTN cases was 1.2 and 1.5 cm and papillary carcinoma 2.0 cm.

**Figure 1:**
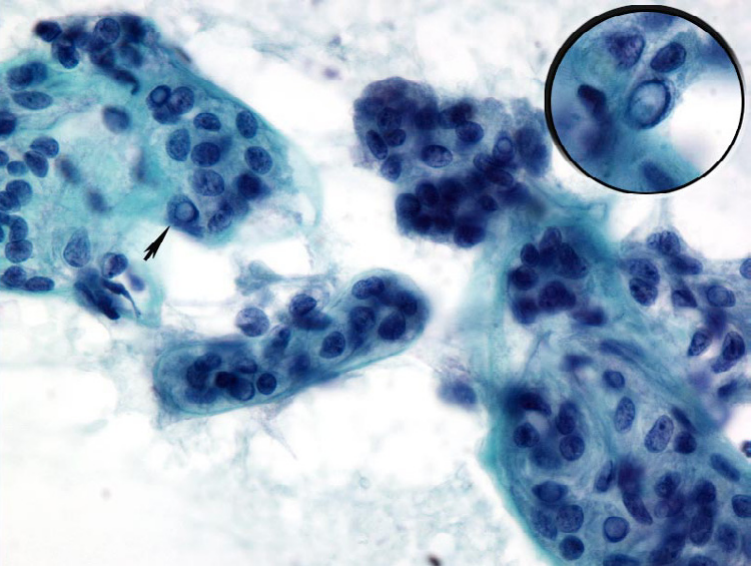
**Cytology of HTN**. Papanicolaou stained fine-needle aspiration of hyalinizing trabecular neoplasm showing elongated tumor enveloped by or associated with acellular stroma. The tumor cells demonstrate nuclear features of papillary carcinoma (arrow and inset).

**Figure 2:**
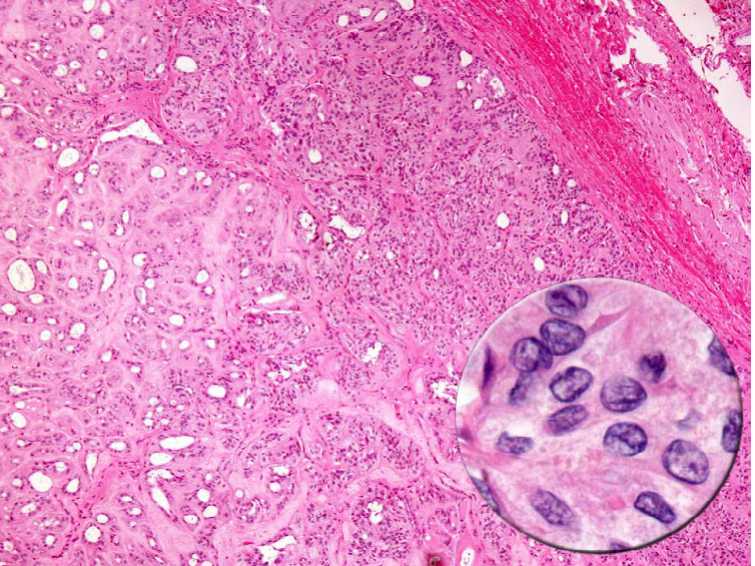
**Cytology of HTN**. Hyalinizing trabecular neoplasm displaying circumscription, encapsulation, tumor cells arranged in nests with intervening hyalinized stroma. The tumor cells demonstrate nuclear features of papillary carcinoma (inset).

In one case of HTN fine-needle aspiration (FNA) was performed under ultrasound guidance. The FNA slides showed elongated tumor cells with nuclear features of papillary carcinoma embedded in an acellular stroma. [Fig 1A] The surgical pathology slides of both cases of HTN showed classic histologic features as described by Carney et al.[1] [Fig 2]

**Figure 3:**
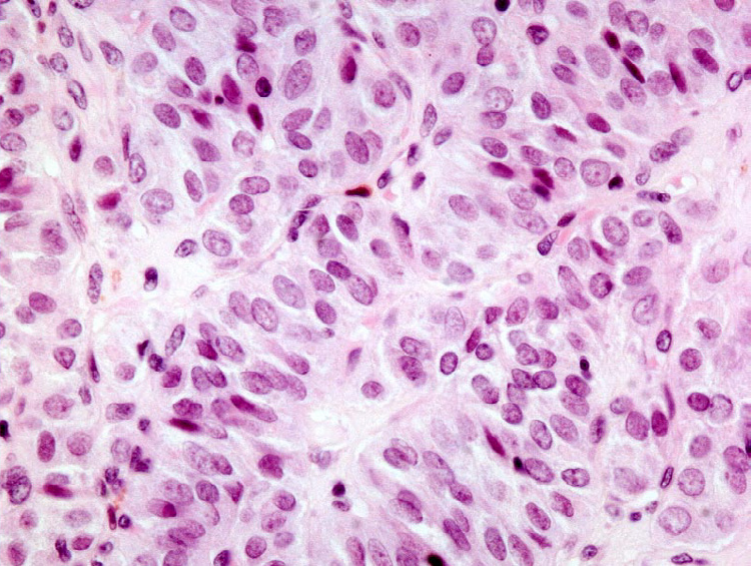
**Histology of HTN**. Trabecular variant of papillary thyroid carcinoma showing trabecular/paraganglioma-like growth pattern.

The case of trabecular variant of papillary carcinoma was comprised of a thinly encapsulated circumscribed tumor showing tumor cells with nuclear features of PTC arranged in trabeculae with minimal sclerosis. There was no evidence of capsular or vascular invasion and the tumor was confined to the thyroid. [Fig 3]

### BRAF mutations

**Figure 4:**
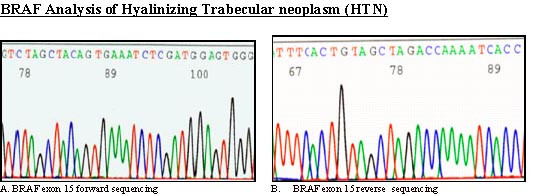
**BRAF analysis**. Sequence chromatogram of hyalinizing trabecular neoplasm with BRAF exon 15 forward primer shows a wild type sequence.

**Figure 5:**
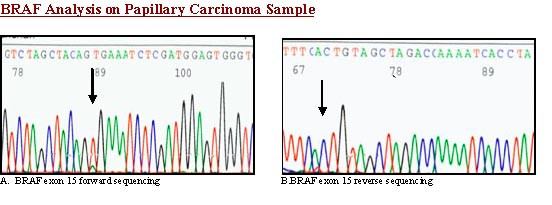
**BRAF analysis**. Sequence chromatogram of trabecular variant of papillary thyroid carcinoma- with BRAF exon 15 forward primer shows BRAF mutation.

BRAF exon 15 was PCR-amplified and subjected to direct sequencing of genomic DNA from all three cases. No BRAF mutations were detected in HTN [Fig. 4]; a heterozygous V600E mutation was found in the case of PTC with trabecular growth pattern [Fig 5].

## Discussion

The debate over whether HTN is a benign tumor or a variant of PTC is ongoing.[7,13] Most experts consider this tumor to be benign based on that all cases of HTN which show classic morphology as described by Carney et al fail to show unequivocal capsular and/or vascular invasion and have not metastasized.[7] However, one cannot totally deny that this is not a variant of PTC due to nuclear cytology, immunoprofile and presence of RET/PTC translocations[10,11,21] In addition, some authors have also suggested that this term should not be used specifically to describe a tumor of thyroid because similar growth pattern can be seen in cases of follicular adenoma, papillary carcinoma and metastatic neuroendocrine tumors to the thyroid gland.[8,14] Because of this debate most experts designate these tumors as hyalinizing trabecular neoplasm.[7,22]

Mitogen Activated Protein Kinase (MAPK) signaling cascades are highly conserved signal transduction pathways that facilitate communication between a cell and its extracellular environment. They coordinate the regulation of a diverse array of gene products, whose expression in turn directs key cellular functions including pathways central to embryogenesis, cell differentiation, proliferation, and death, as well as a number of homeostatic responses of terminally differentiated cells. The Ras-Raf-MEK-ERK MAPK pathway is a particularly well studied MAPK pathway unique to metazoans and consists of a three-tiered kinase cascade from Raf-to-ERK. While activating mutations in the RAS gene family have long been accepted as early events in this process [16], mutations in the B-RAF gene have only recently been recognized for their important contribution to tumor progression Primary malignant melanomas harbor B-RAF mutations at a frequency of 60–67%, while colorectal cancers are affected in approximately 10–12% of all cases sampled. *BRAF *mutation in PTC can range from 29–83% and exclusively occur as the T1799A transversion mutation in exon 15; the mutations in exon 11 (seen in non-thyroidal tumors) are not seen in thyroid tumors.[17,18,23]

In this report we show that cases of HTN which demonstrate classic morphology as described by Carney et al do not demonstrate *BRAF *mutations. The trabecular PTC case showed *BRAF *mutation (heterozygous V600E mutation). As mentioned-above trabecular growth pattern can occur in other thyroid tumors such as papillary thyroid carcinoma, however, in our experience these cases lack the distinct hyalinized stroma enveloping individual tumor cells as seen in HTN. Based on our and other studies [17,24] which have shown absence of BRAF mutations in HTN one can assume that this tumor is not a variant of PTC. However, the caution has to be exercised in confirming the benign nature of this tumor on the basis of absence of BRAF mutation. Since other molecular markers such as RET/PTC and Galectin-3 which were initially thought to be only expressed in PTC are now seen in some benign lesions of the thyroid.[25] Therefore, at present it is advisable to diagnose these tumors as HTN and relay to clinicians that this tumor behaves in benign fashion.

## Competing interests

The author(s) declare that they have no competing interests.

## Authors' contributions

ZWB conceived and designed the study, supervised the research team and data analysis by KP and MB, and wrote the report with KP, MB and VAL. All authors were involved in the critical revision of the manuscript drafts and approved the final version for publication.
